# Supplementary material for: Patterns of Intron Gain and Loss in Fungi
Source: PLoS Biol. 2004 Nov 30;2(12):e422. doi: 10.1371/journal.pbio.0020422 (PMC532390; doi:10.1371/journal.pbio.0020422)
Supplement: Table S1 — Also available at http://genes.mit.edu/NielsenEtAl/. (4.3 MB ZIP). [file pbio.0020422.st001.zip › NielsenEtAl/html/135.html]

AN2577.1.NCU09138.1.MG04839.1.FG04688.1


```
 CLUSTAL W (1.82) Multiple Sequence Alignments - Introns Inserted


Sequence 1: NCU09138.1	310 aa
Sequence 2: MG04839.1	310 aa
Sequence 3: FG04688.1	309 aa
Sequence 4: AN2577.1	316 aa
Alignment Length: 318 aa
Number Identitical Residues: 146 aa
Alignment Score (without introns) 7915


MG04839.1 	MSGPGVGFEYPPTEVSWMKRDVLLFANSIGCKSDELHFLY0ELHPDFAVFPTYPVVLP1F
NCU09138.1	MPGPGVGFEYPPKSVSWCKRDLLLFAQSIGCKAEELHFLY~ELHPNFVPFPTYPLALS1F
FG04688.1 	MSGPGVGFEYSPVEVKWLQRDVLLFANTIGATVDELHFLY0EFDPKFAVFPTYPVILP1F
AN2577.1  	MSGPGAGHEFPAKEVSWLKRDVLLFANSIGATSKELHFLF0ELHPNFAVFPTYPIILP1F
          	*.***.*.*:.. .*.* :**:****::**.. .*****: *:.*.*. *****: *. *

MG04839.1 	KTNTQEVIDFYAAQKSVTIPGVPEFDPTRVVDGQRRIEFLRPLPATSAGKRFEARTKVLG
NCU09138.1	KLDSSDVVDFYAAQKSIAIPGVPVFDPARVVDGQRRIEFFKQLPTSSEGKRFESRTKVVG
FG04688.1 	KKDSQEVVDYYKASKAVQIPGIPKLDYTRVVDGQRKIEFLKALPTTSAGRKFEIRQKVIG
AN2577.1  	KLTDQEVTDFYARSKSSPIPGVPSFDPKRVVDGQRKITVYKQLPTTSAGRKFELRNKVVG
          	*   .:* *:*  .*:  ***:* :*  *******:* . : **::* *::** * **:*

MG04839.1 	VYDKGRPGTVVETQTDLVDAGTGDVYTRATGSAFYVAQGNWGGPKG~PATVNYPPPKGKT
NCU09138.1	VYDKGRPGSVVETQTDIVDATNNEVYSRIHTSSFYVNQGNWGGPKG~PATQNFPPPKDKK
FG04688.1 	VYDKGRPGTVLETETNLVDVESGEVYTRVISSGFFIGQGNWGGPKG~PATKNYPPPEGKA
AN2577.1  	VYDKGK-ASVVETEQTIVDKESGEVYASVVGSAFYVGQGGWGGPKG1PATPNYPPPKDRR
          	*****: .:*:**:  :**  ..:**:    *.*:: **.****** *** *:***:.: 

MG04839.1 	PDAVLEDATTLETPLLYR~LNGDYNPLHATPEPGAKMGFGGVIVHGLWQWNSTCHQLLRH
NCU09138.1	PDAVFENQTTPETPLLYR~LNGDYNPLHADPEPGKKMGFGGVIIHGLYSWNWACHGLLQH
FG04688.1 	PDATIEVQTTPESALLYR~LNGDYNPLHADPAPGQKMGFGGTIMHGLYSWNSTAHALLKE
AN2577.1  	PDAVREIQTTTETAQLYR2LNGDYNPLHATPEPGQKMGFGGIIIHGLYSWNAAAHIVLEA
          	***. *  ** *:. *** ********** * ** ****** *:***:.** :.* :*. 

MG04839.1 	LGGSDPRNIKEYQARFASPVRPGVKLATSVWRTGKVEDGFEEII~-----FETKVVGGKT
NCU09138.1	LGGSDPANIKEYQARFASPIRGGDKLVASAWKTGEIKDGWEEIR~-----FQVQIEGGKV
FG04688.1 	LGGGDPANIKEYQARFPSPVRPGDKLVTHAWVMGEKQGEWSEVR~-----FVTQVAGGKV
AN2577.1  	FGNSDPANFREFQARFASPVKPGDKLITELWRTGEVIEGFEEIR2GMKVSYTLGTAPG-L
          	:*..** *::*:****.**:: * ** :  *  *:    :.*:  . . ::      *  

MG04839.1 	CLSNGRALIKVVGPAKGAKL---
NCU09138.1	VLSNGRALMKCVGPAPHSKL---
FG04688.1 	VLSNGLALMKVTSQGK-SKL---
AN2577.1  	GLSAGDHPHYCVKPAILLQPTKG
          	 ** *      .  .   : :..
```
